# Superior mesenteric vein to the right testicular vein shunt operation for jejunal varices bleeding associated with extrahepatic portal vein obstruction after pancreaticoduodenectomy: a case report

**DOI:** 10.1186/s40792-022-01390-0

**Published:** 2022-02-24

**Authors:** Shohei Shiozaki, Yasuhiro Matsugu, Michinori Hamaoka, Tatsuro Ishimoto

**Affiliations:** 1 Department of Gastroenterological, Breast and Transplant Surgery, Hiroshima Prefectural Hospital, 1-5-54, Ujina-Kanda, Minami-ku, Hiroshima, 734-8530 Japan; 2 Department of Clinical Nutrition, Hiroshima Prefectural Hospital, Hiroshima, Japan

**Keywords:** Portal vein obstruction, Pancreaticoduodenectomy, Testicular vein, Shunt operation, Variceal bleeding

## Abstract

**Background:**

Causes of extrahepatic portal vein obstruction include abdominal surgeries such as pancreaticoduodenectomy. We improved jejunal variceal bleeding due to extrahepatic portal vein occlusion after pancreaticoduodenectomy, by shunting of the testicular vein.

**Case presentation:**

A 72-year-old man was diagnosed with extrahepatic bile duct cancer and underwent subtotal stomach-preserving pancreaticoduodenectomy 5 years ago. No postoperative complications occurred, adjuvant chemotherapy using gemcitabine hydrochloride was performed, and the patient remained recurrence-free. One year and 6 months post-operation, extrahepatic portal vein stenosis appeared, but no recurrence was noted. However, 4 years and 6 months later, recurrent gastrointestinal bleeding occurred, and the patient was diagnosed with an extrahepatic portal vein obstruction. Double-balloon enteroscopy showed capillary dilatation and varicose veins in the hepaticojejunostomy region, and venous bleeding from collateral blood vessels was diagnosed. A superior mesenteric vein to the right testicular vein shunt operation was performed, following which the gastrointestinal bleeding disappeared, and the anemia improved. Although transient hepatic encephalopathy occurred, conservative treatment relieved it. Double-balloon enteroscopy confirmed the disappearance of abnormal blood vessels.

**Conclusions:**

A portosystemic shunt operation using the right testicular vein effectively relieved refractory variceal bleeding around the hepaticojejunostomy site in the jejunum due to an extrahepatic portal vein obstruction after pancreaticoduodenectomy.

## Background

Extrahepatic portal vein obstruction is a rare condition, but its frequency is expected to increase with the long-term survival of patients undergoing pancreaticoduodenectomy (PD) [1]. The leading causes of extrahepatic portal vein obstruction after PD’s are periportal inflammation associated with portal vein thrombi, lymphadenectomy, postoperative pancreatic fistula, and cancer recurrence. In this situation, collateral blood vessels and varicose veins tend to develop around the hepaticojejunostomy region in the jejunal rim, which causes massive gastrointestinal bleeding with portal hypertension, and portal decompression is necessary for hemostasis [2]. Recently, there have been increasing reports of minimally invasive treatments for portal vein obstruction after PD, such as portal vein stenting and balloon dilation using vascular interventional radiology (IVR). However, their indications and long-term patency remain under debate [3, 4]. A portosystemic shunt operation can provide reliable portal decompression; alternatively, control of hepatic encephalopathy is required. We report a successful case of a portosystemic shunt operation using the right testicular vein as a bypass vessel in a patient with extrahepatic portal vein obstruction and refractory gastrointestinal bleeding after PD.

## Case presentation

**Fig. 1:**
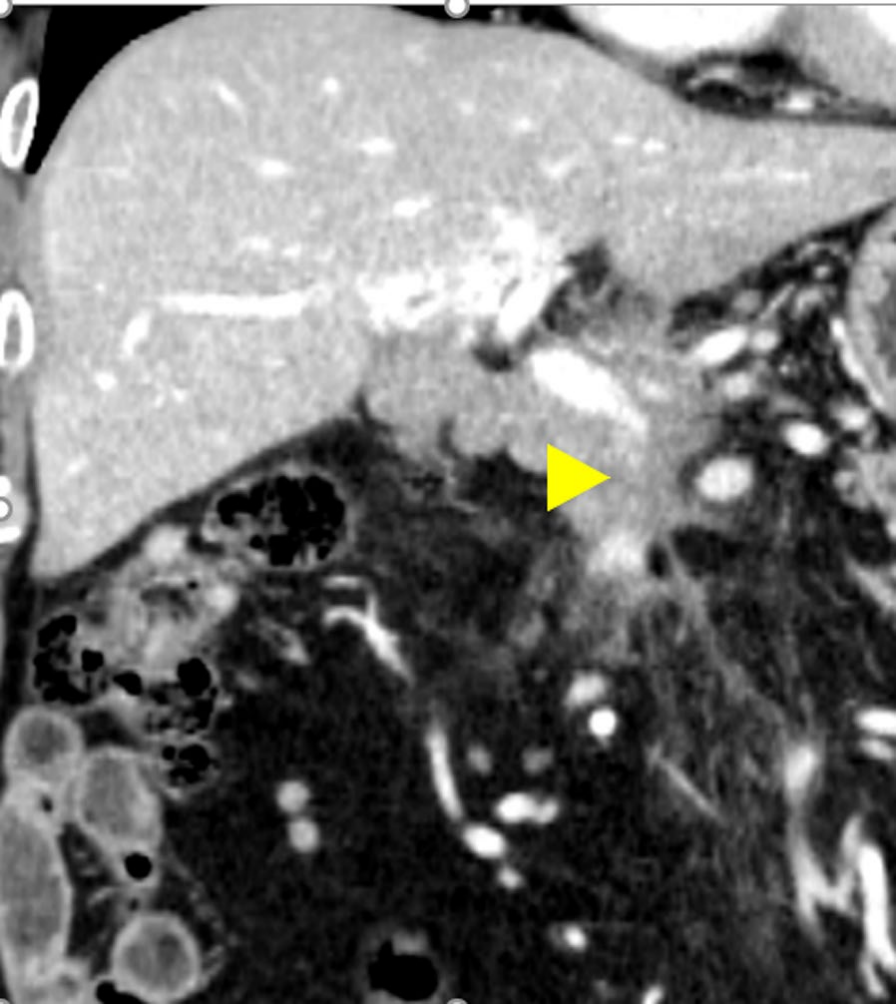
Follow-up computed tomography a year and 6 months after pancreaticoduodenectomy (coronal image). The superior mesenteric vein was obstructed near the splenic vein confluence (arrowhead)

A 72-year-old man reported refractory melena. He had undergone a subtotal stomach-preserving PD with standard lymphadenectomy, for extrahepatic bile duct cancer 5 years previously. The operating time was 602 min, and the amount of bleeding was 908 mL for extrahepatic bile duct cancer 5 years previously. Due to the suspected invasion of the transverse colon and gallbladder bed, a partial resection of the transverse colon and gallbladder bed was performed simultaneously. Neither the transverse colon nor the gallbladder bed yielded malignant findings. Grade A postoperative pancreatic fistula was observed after surgery (drain amylase 3 days after the surgery: 6113 U/L) [5]. The histopathological diagnosis was pT3N0M0, pStage IIB [6] with negative resection margins. He received six courses of gemcitabine hydrochloride as adjuvant chemotherapy and remained recurrence-free. A year and 6 months after the operation, follow-up contrast-enhanced computed tomography (CT) revealed extrahepatic portal vein stenosis (Fig. 1). However, the patient was not symptomatic, and there was no recurrence. Later, he experienced anaphylactic shock and cardiac arrest due to an allergic reaction to the iodine contrast medium and a contrast-enhanced CT scan could not be performed. Four years and 6 months later, recurrent gastrointestinal bleeding had occurred, the frequency of melena worsened, and blood transfusions were required. Blood tests showed severe anemia, but hepatobiliary enzyme levels and tumor markers were within normal ranges (Table 1).

**Table 1:** Blood test

White blood cell, μL	6200
Hemoglobin, g/dL	7.2
Platelet, μL	319,000
C-reactive protein, mg/dL	1.79
Aspartate aminotransferase, U/L	31
Alanine transaminase, U/L	42
Alkaline phosphatase, U/L	595
Total-bilirubin, mg/dL	0.5
Lactate dehydrogenase, U/L	226
NH_3_, µg/dL	55
Albumin, g/dL	2.7
Blood urea nitrogen, mg/dL	8.9
Creatinine, mg/dL	0.75
Carcinoembryonic antigen, ng/mL	3.3
Carbohydrate antigen 19-9, U/mL	13
Prothrombin time-international normalized ratio	1.03
Activated partial thromboplastin time, min	30.5

Blood tests showed severe anemia, but hepatobiliary enzyme levels and tumor markers were within normal ranges

**Fig. 2:**
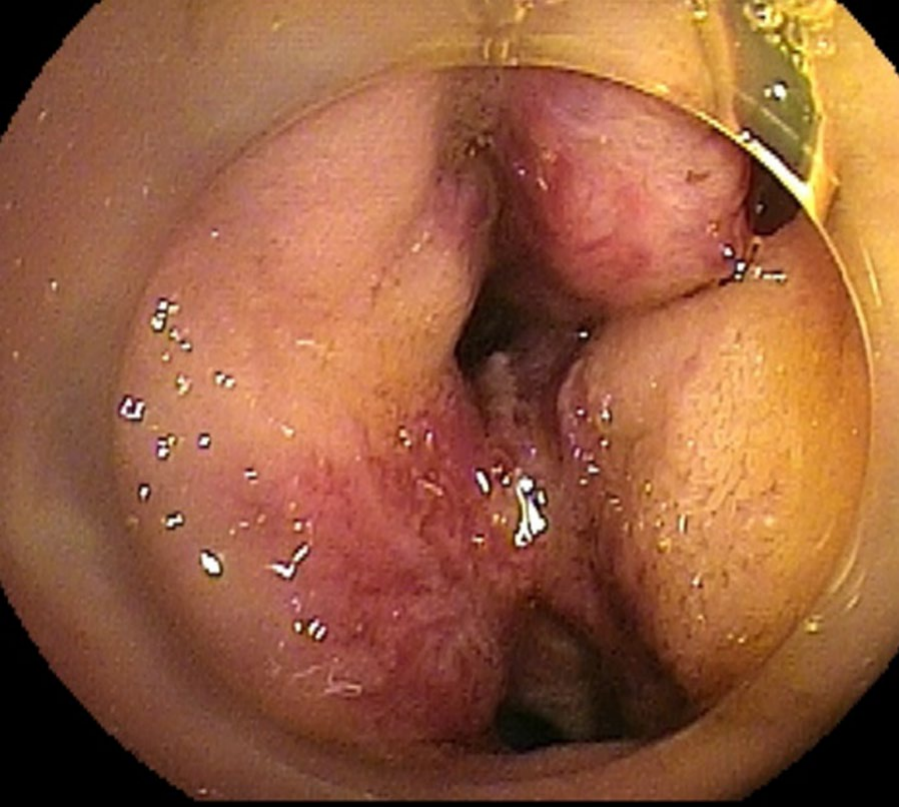
Preoperative findings of double-balloon enteroscopy. Capillary and venous dilatation of the mucosa near the hepaticojejunostomy site and varicose vein elevation were detected

**Fig. 3:**
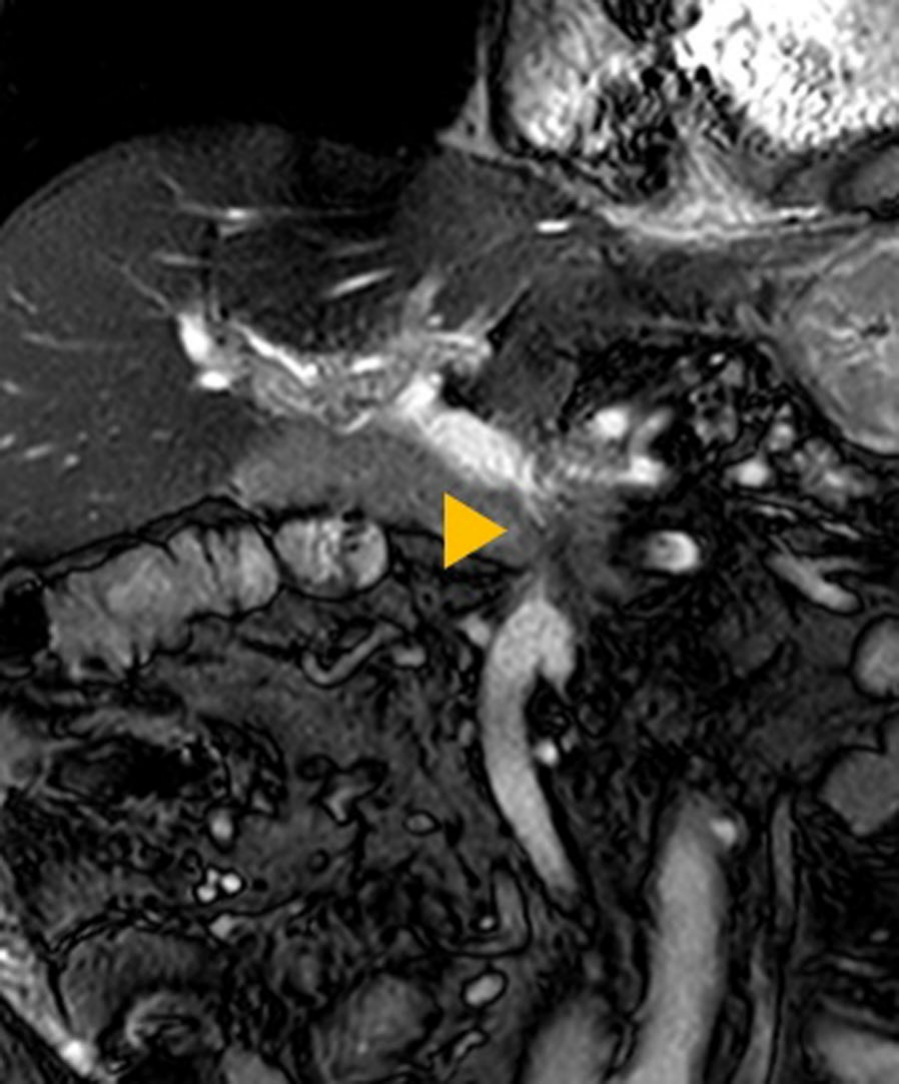
Preoperative findings of contrast-enhanced Magnetic Resonance Imaging (coronal image). The superior mesenteric vein was obstructed 2 cm near the splenic vein confluence (arrowhead), and the splenic vein was stenotic

Upper gastrointestinal enteroscopy revealed no apparent bleeding lesions in the esophagus or stomach. Double-balloon (DB) enteroscopy showed capillary–venous dilatation and varicose veins around the hepaticojejunostomy region in the jejunum, which was diagnosed as developed collateral blood vessels due to postoperative portal hypertension (Fig. 2). Total colonoscopy did not reveal any obvious bleeding source, and bloody bowel discharge was observed in the ileum. Contrast-enhanced magnetic resonance imaging (MRI) showed that the superior mesenteric vein (SMV) was obstructed by 2 cm around the splenic vein confluence (Fig. 3). The left gastric vein and the hilar collateral vessels were well developed, and the right testicular vein had no anatomical abnormalities and flowed into the inferior vena cava (IVC). Neither positron emission tomography nor CT showed a significant periportal accumulation (SUVmax: 1.6), and cancer recurrence was not suspected. We diagnosed that the portal vein occlusion caused an afferent collateral blood channel to develop, which led to varicose veins around the hepaticojejunostomy region, resulting in venous hemorrhage.

**Fig. 4:**
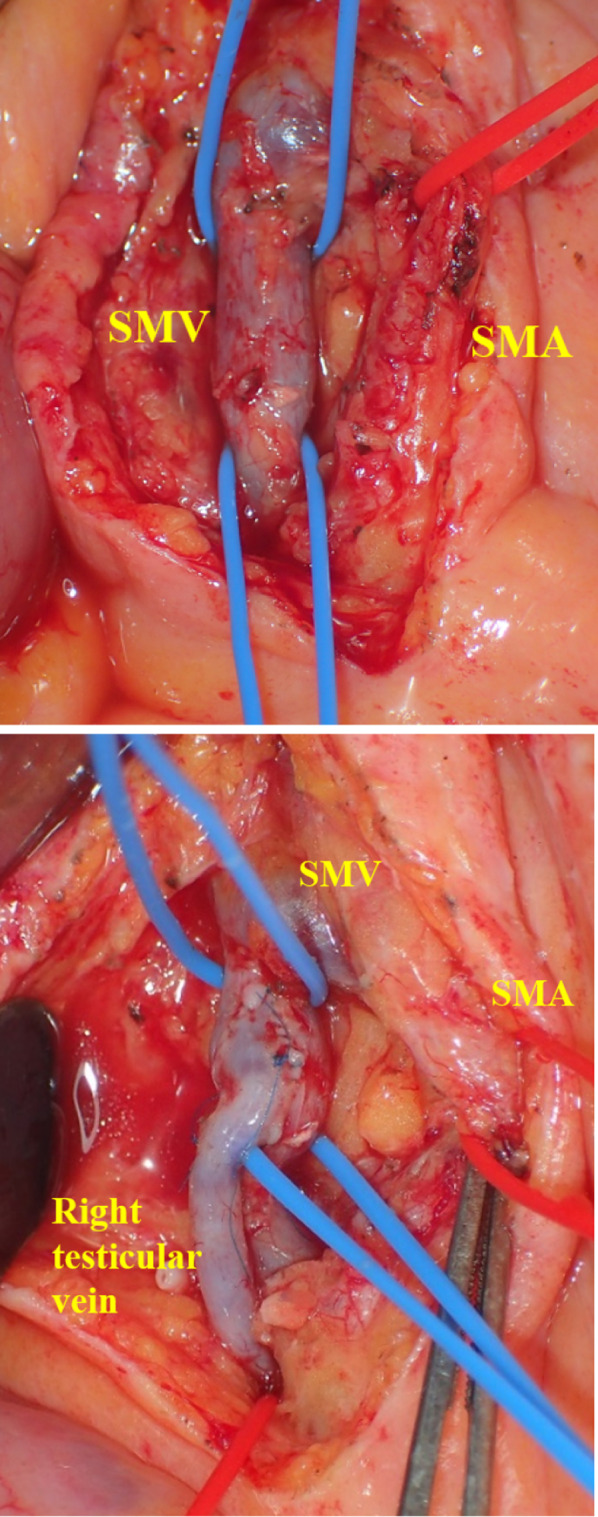
Operative findings. We performed an end-to-side anastomosis between the right testicular vein and superior mesenteric vein. Dilatation of the right testicular vein was healthy after the anastomosis

**Fig. 5:**
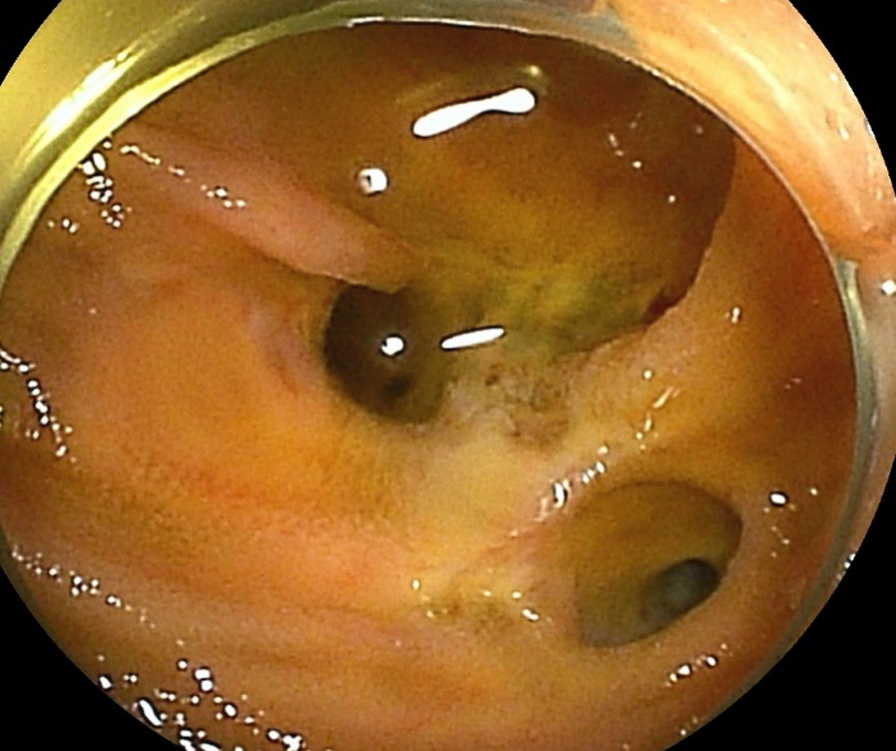
Findings of double-balloon enteroscopy 1 month after surgery. Capillary and venous dilation of the mucosa near the hepaticojejunostomy site disappeared

**Fig. 6:**
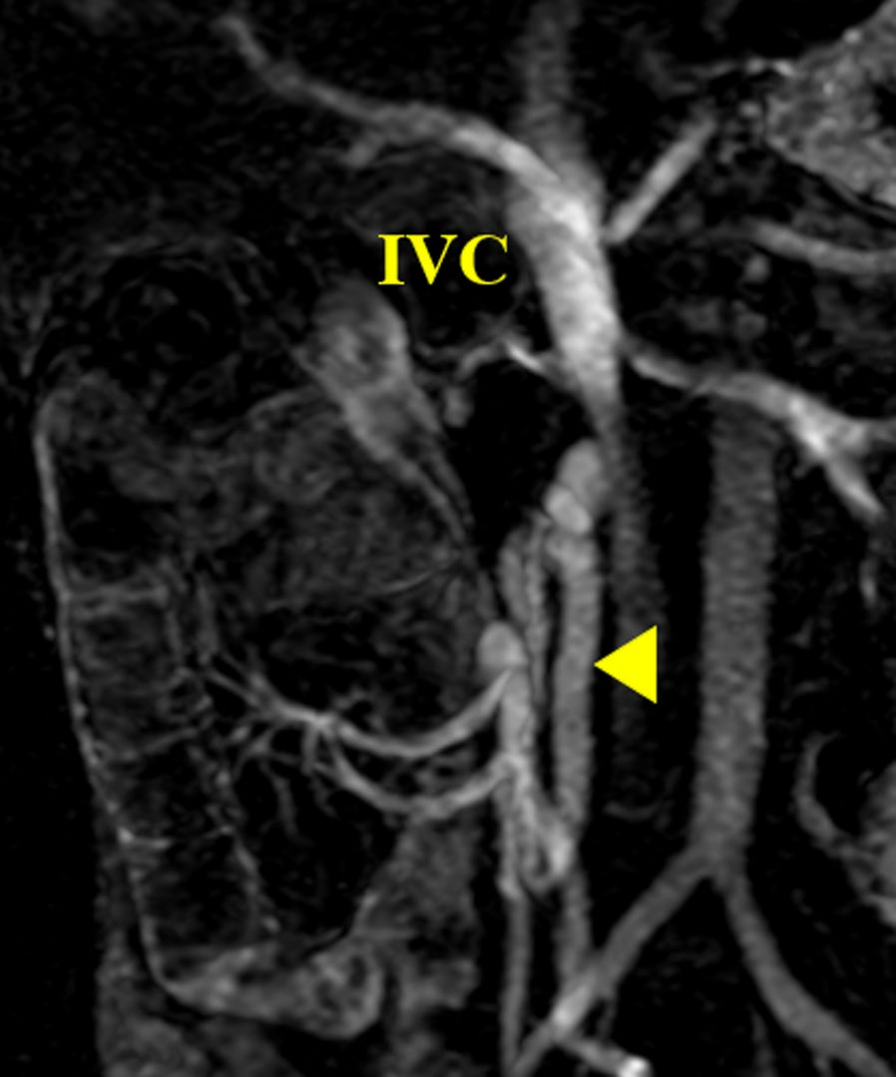
Postoperative findings of contrast-enhanced magnetic resonance imaging (coronal image). Blood flow in the right testicular vein is maintained (arrowhead) and the shunt is open

Because of severe iodine allergy history, IVR treatment would be complex, and therefore, we chose a portosystemic shunt operation. A midline incision was made, and serous ascites were found in the abdominal cavity. The small intestine and mesentery were markedly telangiectatic, suggesting SMV congestion. A branch of the SMV in the mesentery at the end of the ileum was cut down, and a venous catheter was inserted to measure the SMV pressure, which was as high as 33 mmHg. Subsequently, the right testicular vein was identified and dissected at the IVC junction. The SMV and superior mesenteric artery near the small bowel mesenteric root were dissected and taped. The testicular vein penetrating the mesentery was anastomosed with SMV (Fig. 4). The testicular vein was well dilated after anastomosis, and the SMV pressure dropped from 33 to 22 mmHg. Furthermore, we confirmed steady blood flow from the SMV to the testicular vein using Doppler ultrasonography (19.1 cm/s). On postoperative day three, blood ammonia levels increased to 265 mcg/dL, and the patient developed hepatic encephalopathy. With oral rifaximin and defecation control with lactulose, blood ammonia levels normalized, and the symptoms disappeared. Subsequently, oral warfarin was administered, and the patient was discharged on postoperative day 15. One month after surgery, DB enteroscopy confirmed the disappearance of capillary and venous dilatation of the mucosa around the hepaticojejunostomy region (Fig. 5). At a year and 6 months post-operation, contrast-enhanced MRI confirmed that the testicular vein as a bypass between the SMV and IVC was working well (Fig. 6), and blood ammonia levels were within reference ranges without medication.

## Discussion

Extrahepatic portal vein occlusion is classified as primary or secondary, and causes such as abdominal surgery, like PD, are secondary. The causes of extrahepatic portal vein obstruction after PD include postoperative portal vein thrombosis, periportal adhesions, inflammation due to lymph node dissection or anastomotic leakage, and tumor recurrence around the portal vein [2]. One report noted that a postoperative pancreatic fistula was an independent risk factor for benign PV stenosis after PD [7]. In this case, there was a grade A pancreatic fistula after PD surgery, which was considered as a cause of the portal vein stenosis. As long-term survival after PD has become possible [1], late complications include varicose vein formation and gastrointestinal bleeding due to stenosis or occlusion of the portal vein.

Ectopic variceal bleeding is generally massive and life-threatening with a mortality rate of approximately 40%. Up to 17% of ectopic varices occur in the duodenum [8]. Varicose veins of the jejunum after PD are often challenging to reach using an endoscope, making diagnosis difficult. Additionally, endoscopic hemostasis is difficult. However, DB enteroscopy has recently made it possible to visualize and diagnose varicose veins [9]. Here, we identified vasodilatation near the hepaticojejunostomy site using DB enteroscopy resulting in the diagnosis.

Endoscopic treatment of varices of the jejunum in the presence of portal vein stenosis causes hepatofugal portal venous flow, resulting in the exacerbation of esophagogastric varices and bleeding. Repeated bleeding from the jejunum and gastroesophageal varices after endoscopic treatment of portal vein stenosis patients have been reported [10]. Unlike esophageal varices in cirrhosis, the endoscopic hemostatic effect of hepatopetal jejunal varices due to extrahepatic portal vein obstruction is short-lived, and other means of hemostasis are required. Therefore, it is important to treat portal stenosis, which is the essence of the disease, and to lower portal pressure as a fundamental treatment procedure. Recently, an increasing number of reports have noted that portal vein stenting and transjugular intrahepatic portosystemic shunt (TIPS) are less invasive, more physiological, and more practical than revascularization. Portal vein stenting has improved clinical symptoms in over 80% of cases [3, 11]. However, there have been some reports of patients experiencing gastrointestinal bleeding after stenting, and the average patency period has been reported as 6 days to 29 months [12], which is not long enough for long-term patency. TIPS was not applicable in this case because of extrahepatic portal vein obstruction.

Surgical revascularization includes a Rex shunt and a portosystemic shunt. In the portosystemic shunt, the shunting site and anastomosis method can be selected according to the situation [10]. The efficacy of the portosystemic shunt for varicose veins has been reported to be 100% while the efficacy of the Rex shunt has been reported to be 96% [13]. The Rex shunt for adults with severe adhesions due to PD has been less common because of its difficulty and complexity [14]. A spleno-renal or inferior mesenteric–renal vein shunt was not recommended due to stenosis of the splenic and inferior mesenteric veins [15]. Therefore, we performed a portosystemic shunt (SMV–IVC) using a testicular vein as the shunt vessel without a graft. To our knowledge, this is the first case of a portosystemic shunt using the testicular vein. This shunting procedure is performed caudal to the transverse mesentery, so postoperative adhesions were not a concern. Additionally, the right testicular vein ran dorsal to the small mesentery, allowing for a relatively long neurovascular pedicle that could be freely anastomosed with the SMV. Moreover, it has been reported that distal splenic renal shunting for portal vein stenosis preserved hepatic portal blood flow in 88–90% of cases in the early postoperative period, suggesting that portal blood flow is less affected and the risk of hepatic encephalopathy is lower than for major shunting operations, such as a portal–IVC shunt [16, 17]. In this case, portal pressure was maintained at 22 mmHg after the bypass surgery, suggesting that the portosystemic shunt using the testicular vein worked similarly to a selective shunting procedure such as a distal spleno-renal shunt. Therefore, it is considered to have a lower risk of hepatic encephalopathy than significant shunts.

## Conclusions

Although the postoperative follow-up period is still short in this case, a portosystemic shunt using the testicular vein was suggested as an effective treatment for varicose veins of the jejunum after PD. More cases must be reviewed, conducted, and examined to validate the research findings of the study and improve future treatment designs.

## Data Availability

Not applicable.

## Abbreviations

PD: Pancreaticoduodenectomy
IVR: Vascular interventional radiology
CT: Computed tomography
DB: Double-balloon
MRI: Magnetic resonance imaging
SMV: Superior mesenteric vein
IVC: Inferior vena cava
TIPS: Transjugular intrahepatic portosystemic shunt
